# Correction: Medication adherence and quality of life among geriatric patients: Insights from a hospital-based cross-sectional study in India

**DOI:** 10.1371/journal.pone.0325215

**Published:** 2025-05-27

**Authors:** Umaima Farheen Khaiser, Rokeya Sultana, Ranajit Das, Saeed G. Alzahrani, Shahabe Saquib, Shaheen Shamsuddin, Mohammad Fareed

The following information is missing from the Funding statement: The authors extend their appreciation to the Deanship of Scientific Research and Graduate Studies at King Khalid University for supporting this work through Large group Project RGP-2/504/45.
