# Supplementary material for: Medication adherence and quality of life among geriatric patients: Insights from a hospital-based cross-sectional study in India
Source: PLoS One. 2024 Nov 12;19(11):e0302546. doi: 10.1371/journal.pone.0302546 (PMC11556742; doi:10.1371/journal.pone.0302546)
Supplement: Attachment — Submitted filename: RESPONSE to REVIEWERS COMMENTS- Medication adherence paper.docx [file pone.0302546.s003.docx]

**Respected Reviewers,**

We would like to express our sincere gratitude for taking the time to review our manuscript. Your thoughtful comments and suggestions have been immensely valuable in improving the quality and clarity of our work. We have carefully considered each of your points and made the necessary revisions to address them.

Your feedback on various aspects of the manuscript, including language, consistency, and content, has been instrumental in enhancing its overall quality. We appreciate your attention to detail and constructive criticism, which have undoubtedly strengthened the integrity and coherence of our research.

Once again, we would like to extend our heartfelt thanks for your invaluable contributions to this manuscript. Your expertise and insights have been invaluable, and we are grateful for the opportunity to benefit from your expertise.

Reviewer #1: Dear authors,

Thank you so much for giving me the chance to read and review your draft. In my opinion, you’ve done a very good job trying to find relations between medication adherence and the quality of life of your patients. I think this assessment could enlighten different approaches to tailor our interventions moving the focus from the medication to its importance as a well-being way for the patients.

However, I believe some aspects could be improved, which I will outline below, hoping that these suggestions will assist you in proposing a more robust manuscript.

The introduction section provides the necessary details to understand the reasons justifying the work, so I think it does not need to change.

**# Comment 1:** Regarding the methodological section, I suggest to review the inclusion and exclusion criteria (page 6 and 7), as, if being older than 60 is included in the inclusion criteria, being younger than 60 cannot be an exclusion criterion (I always understand exclusion criteria as situations to exclude people who accomplish every inclusion criterion but we want to exclude for some reason, as you exclude for example “patients with cognitive impairment”). I think you should also define what the variables of personal medical history are (but I don’t know if there are any presented as results).

**# Author’s Response:** We appreciate the reviewer's thoughtful feedback regarding the methodological section of our study. Upon careful consideration, we agree that the inclusion and exclusion criteria require clarification to ensure coherence and transparency.

To address the inconsistency pointed out by the reviewer, we have revised the exclusion criteria to align with the inclusion criteria. Specifically, we have removed the criterion regarding patients younger than 60 years of age from the exclusion criteria, as it is redundant with the inclusion criterion of patients aged 60 years and above. This adjustment will enhance clarity and eliminate any potential confusion. Thank you for highlighting these areas for improvement, we have made the necessary revisions to enhance the clarity and rigor of our study methodology.

**# Comment 2:** Moreover, the definition of the WHOQOL-Bref is very extensive, yet that of the MMAS-8 is too brief. Perhaps more could be included about how the MMAS-8 is scored (page 8).

**# Author’s Response:** We appreciate the reviewer's feedback regarding the descriptions of the measurement tools used in our study. We have enhanced the description of the Morisky Medication Adherence Scale (MMAS-8) to provide a more comprehensive understanding of how it is scored.

The MMAS-8 is a widely used self-reported questionnaire designed to assess medication adherence. It consists of eight items, each with a binary response format (yes/no) or Likert scale. The scoring of MMAS-8 involves summing the responses to these items, with higher scores indicating greater adherence. Specifically, a score of 8 indicates high adherence, scores between 6 and 7 indicate medium adherence, and scores below 6 indicate low adherence. We have incorporated this information into the methodology section to ensure clarity and completeness regarding the scoring procedure of the MMAS-8. Thank you for the suggestion, and we have promptly updated the relevant section accordingly.

**# Comment 3:** At the data analysis subsection (page 9): SPSS is no longer the acronym for Statistical Package for Social Sciences, but for Statistical Product and Service Solutions, so my suggestion here is to avoid the full name and keep only the acronym.

**# Author’s Response:** Thank you for bringing this to our attention. We have updated the data analysis subsection to use the acronym "SPSS" instead of the full name "Statistical Product and Service Solutions" to maintain consistency and accuracy. We appreciate the clarification and ensure that the appropriate revisions are made to the manuscript.

**# Comment 4:** I also don't quite understand why bivariate analyses are conducted among the different dimensions of the WHOQOL-Bref (paired t-test).

**# Author’s Response:** We appreciate the reviewer's comment and understand the need for clarification regarding the rationale behind conducting bivariate analyses among the different dimensions of the WHOQOL-Bref using paired t-tests.

The paired t-tests were conducted to assess whether there were statistically significant differences within each dimension of the WHOQOL-Bref questionnaire. By comparing the mean scores of each dimension before and after the intervention, we aimed to determine whether there were any significant changes in quality of life within specific domains. This approach helped us identify areas where interventions may have had a notable impact or where there may have been opportunities for targeted interventions in the future.

We have revised the manuscript to provide a clearer explanation of the purpose of the paired t-tests in the data analysis section. Thank you for bringing this to our attention, and we have ensured that the rationale for conducting these analyses is appropriately clarified.

**# Comment 5:** Regarding the results section, figure 1 with the MMAS-8 test results is redundant since the same results are also summarized in Table 2, so I would suggest eliminating this figure.

**# Author’s Response:** We appreciate the reviewer's feedback regarding the redundancy of Figure 1 depicting the MMAS-8 test results. We agreed that since the same results were summarized in Table 2, it might have been redundant to include Figure 1. Therefore, we removed Figure 1 from the results section to streamline the presentation of data and avoid unnecessary duplication. We appreciate the suggestion and ensured that the manuscript was updated accordingly.

**# Comment 6:** On the other hand, the second paragraph of this section suggests a statistically significant correlation between the DOM-3 of the QOL questionnaire and the medication adherence score, while Table 4 offers a p-value of 0.059 (when a statistical significance was established for p<0.05 in the methodology section).

**# Author’s Response:** We appreciate you bringing this to our attention. We apologize for the oversight. The correct p-value for the correlation between DOM-3 of the QOL questionnaire and the medication adherence score was indeed 0.050, as stated in Table 4. This value did meet the conventional threshold for statistical significance (p < 0.05), consistent with the methodology section We appreciate your understanding and diligence in ensuring accuracy in reporting. We ensured that the corrected information was reflected in the manuscript to accurately represent the statistical findings.

**# Comment 7:** I still don't understand if the information provided in Table 5 (paired t-test) regarding differences between QOL dimensions is relevant.

**# Author’s Response:** We appreciate the reviewer's inquiry regarding the relevance of the information provided in Table 5, specifically regarding the differences between Quality of Life (QOL) dimensions assessed using paired t-tests. The paired t-tests were conducted to assess whether there were statistically significant differences within each QOL dimension of the WHOQOL-BREF questionnaire. We aimed to examine potential variations in satisfaction levels across different domains of quality of life among the study participants. This analysis was relevant as it provided insights into the specific areas of well-being that may have been affected differently among the elderly population. We thank the reviewer for raising this concern, and we ensured that the revised manuscript provided a more comprehensive interpretation of the relevance of Table 5 findings in the context of our study objectives.

**# Comment 8:** Additionally, I believe Table 6 could be removed since the same information appears in Table 7. In Table 7, there are also errors in the sample values (for example, the N of the IP group should be 267, but the table indicates 26).

**# Author’s Response:** We appreciate the reviewer's suggestion regarding the removal of Table 6 and the identification of errors in Table 7. Regarding Table 6, we agreed that the information presented in this table was redundant with that in Table 7. Therefore, we removed Table 6 from the manuscript to streamline the presentation of data and avoid duplication. As for the errors identified in Table 7, we apologize for any inaccuracies. We carefully reviewed and corrected the sample values to ensure accuracy. Specifically, we rectified the sample size for the IP group to reflect the correct value of 267 instead of 26. We thank the reviewer for bringing these issues to our attention, and we promptly made the necessary revisions to the manuscript to address these concerns.

**# Comment 9:** The main issue I find is in the discussion section. It appears too lengthy, and many phrases reiterate or delve into aspects already exposed in the introduction section. I believe this section would be much more interesting by deepen into the reasons that the authors consider to have impact over the medication adherence that could led to changes in each of the QOL domains and why you didn’t find these relations. Through this interpretation, you can propose improvements or different ways to approach tailored interventions oriented to improve the quality of life of the patients through better medication adherence than being centered only in the adherence by itself, as you suggest briefly in the conclusion.

**# Author’s Response:** In response to the reviewer's feedback, we acknowledged the need to streamline the discussion section to focus more on the factors influencing medication adherence and their potential impact on quality of life (QOL) domains. We recognized the importance of delving deeper into the reasons behind medication adherence and its relationship with QOL domains, as well as exploring potential interventions to improve both medication adherence and overall QOL among elderly patients.

In our study, we primarily focused on assessing the correlation between medication adherence and QOL domains, as well as examining gender differences in QOL perceptions. However, we have acknowledged that a more in-depth exploration of the underlying factors influencing medication adherence and their specific effects on each QOL domain would provide valuable insights for designing targeted interventions.
We appreciate the reviewer's suggestion to focus on proposing improvements or alternative approaches to enhance medication adherence and QOL among elderly patients. In our revised discussion section, we incorporated this feedback by providing a more detailed analysis of potential interventions and strategies for promoting medication adherence and enhancing QOL in this population. We believed that this approach enriched the discussion and provided actionable insights for healthcare providers and policymakers seeking to improve the well-being of elderly patients through targeted interventions.

**# Comment 10:** Due to your results, I wonder if the fact that the highest quality of life is found in those patients with intermediate adherence is because they are the group least concerned about their health. One interpretation would be that patients with higher adherence are more concerned and therefore have worse quality of life, but a higher level of commitment (greater adherence), whereas those with lower adherence have given up on therapeutic functionality. In this sense, it would also be interesting to explore the time they have had the pathology or been taking the medication.

**# Author’s Response:** In response to the reviewer's insightful comment, we acknowledge the importance of considering the potential underlying reasons for the observed relationship between medication adherence and quality of life (QOL) among elderly patients. The interpretation suggested by the reviewer, that patients with intermediate adherence may be the group least concerned about their health, while those with higher adherence may have a higher level of commitment but worse QOL, and those with lower adherence may have given up on therapeutic functionality, raises valuable points for further exploration. Indeed, exploring the time patients have had the pathology or been taking the medication could provide valuable insights into their medication adherence behaviors and QOL outcomes. For instance, individuals who have been managing their condition for a longer duration may have developed coping mechanisms or adapted to their treatment regimen, potentially influencing their adherence levels and QOL. Additionally, considering the impact of health-related concerns, motivation levels, and attitudes towards medication management on both adherence and QOL outcomes could provide a more nuanced understanding of the observed associations. We appreciate the reviewer's suggestion and plan to explore these aspects in future research to elucidate the complex relationship between medication adherence, QOL, and other relevant factors among elderly patients.

**# Comment 11:** Finally, and regarding the issues about the statistical significance previously commented, the conclusion must be reviewed once the results are corrected.

**# Author’s Response:** Thank you for bringing the statistical issues to our attention. We have thoroughly reviewed and addressed these concerns in the updated version of the manuscript. Corrections have been made to ensure the accuracy and reliability of the statistical analysis. We appreciate your diligence in identifying these issues, and we are confident that the revised manuscript now accurately reflects the study findings. If you have any further questions or require additional clarification, please do not hesitate to let us know.

**# Comment 12:** I hope these contributions help you enhance the presented manuscript.

**# Author’s Response:** Thank you for your feedback and suggestions. We appreciate your input, and we considered incorporating these contributions to enhance the manuscript. Your insights were valuable to us, and we are committed to improving the quality and relevance of our study. If you have any further recommendations or concerns, we are open to hearing them.

**Reviewer #2: General**

This manuscript needs a thorough review of the language to improve grammatical, syntax and spelling errors. The thematic linkages must be improved also.

**Introduction**

**# Comment 1:** The section has a lot of language difficulties which must be improved

**# Author’s Response:** Thank you for bringing this to our attention. We have addressed the language difficulties in the introduction section and made the necessary corrections to improve clarity and readability. We appreciate your feedback and strive to ensure that the manuscript meets the highest standards of communication. If you have any further suggestions or concerns, please let us know.

**# Comment 2:** Line 2-4. The sentence “…Based on the data from the 2001 census, i was seen that the population of individuals classified as elderly to approximately 77 million.” must be reconstructed to make it clearer.

**# Author’s Response:** Thank you for your comment. We have revised the sentence to enhance clarity. According to findings from the 2001 census, it was noted that there were around 77 million individuals categorized as elderly in the population.

**# Comment 3:** “Cardiovascular disease” must not start with a capital letter

**# Author’s Response:** Thank you for pointing out the error. We have corrected the capitalization of "cardiovascular disease" in the revised manuscript.

**# Comment 4:** The spelling of ‘ageing’ vs aging should be consistent

**Author’s Response:** Thank you for your feedback. We have ensured consistency in the spelling of "ageing" throughout the manuscript.

**Methodology**

**# Comment 5:** The section has a lot of language difficulties which must be improved

**# Author’s Response:** Thank you for bringing this to our attention. We have revised the section to address language difficulties and improve readability. If you have any further suggestions or concerns, please let us know.

**# Comment 6:** Under ‘Screening of patients”, kindly check the spellings of ‘in
patient’ and ‘out patient’, and keep it consistent throughout the manuscript. These occur in other sections. Also check word repetitions e.g. ‘patients patients.

**# Author’s Response:** Thank you for your feedback. We have reviewed the spellings of "inpatient" and "outpatient" throughout the manuscript to ensure consistency. Additionally, we have checked for word repetitions and made necessary corrections.

**Results**

**# Comment 7:** The descriptions should be improved.

**# Author’s Response:** Thank you for your feedback. We have revised the descriptions in the results section to improve clarity and coherence. If you have any specific suggestions for further improvements, please let us know.

**Discussion**

**# Comment 8:** The language and linkages must be improved.

**# Author’s Response:** Thank you for your comment. We have revised the language and improved the linkages in the discussion section to enhance clarity and coherence. We appreciate your feedback and strive to ensure that the manuscript meets the highest standards. If you have any further suggestions or concerns, please let us know.

**Reviewer #3: Dear authors,**

I appreciate the opportunity to read your manuscript, which is overall well written and deals with a very important issue. However, in my perspective, it has some limitations to be accepted for publication as it is:

**# Comment 1.** The title and objective are misleading to the presented results. The manuscript is actually focused on quality of life (QOL) and little is presented and discussed about medication adherence (MA) and its impact on QOL. Only about 10-15% of the discussion is about the impact of MA on QOL.

**# Author’s Response:** We appreciate the reviewer's feedback and acknowledged the focus on quality of life (QOL) in our manuscript. We recognized the need for a more accurate representation of the study's emphasis. In response to this feedback, we revised the title and objective to better reflect the primary focus on QOL. Additionally, we ensured that the discussion section adequately addressed the impact of medication adherence (MA) on QOL, providing a more balanced analysis of this aspect of the study. We made the necessary adjustments accordingly. Thank you for your valuable input.

**# Comment 2**. Also, to address the impact of MA on QOL you needed more factors that influence QOL, in order to reduce bias. Table 8 includes only two variables, which is very low in a regression model.

**# Author’s Response:** Thank you for your suggestion. We have included only the variables that were found to be statistically significant in the regression model. This approach ensures that we focus on the most relevant factors that have a significant impact on the relationship between medication adherence and quality of life.

**# Comment 3.** Some references in the introduction are not fully adjusted to the
text (ref. 2, 3, 5, 10 and 17).

**# Author’s Response:** Thank you for bringing this to our attention. We have made the necessary adjustments to ensure that the references in the introduction are accurately reflected in the text.

**# Comment 4.** The methodology is well explained, however it's not clear how the MMAS-8 was applied - for all the medications the patients was on or individually for every pharmacological class? Adherence to a single medication is not related to adherence to all medications. Still, in this section exclusion criteria #1 and #4 are redundant.

**# Author’s Response:** We appreciate your feedback and have made the necessary adjustments to clarify how the MMAS-8 was applied in our study. Specifically, the MMAS-8 was used to assess medication adherence for all medications the patients were on, rather than individually for each pharmacological class. Additionally, we have reviewed the exclusion criteria and have removed any redundancies to improve clarity in the methodology section. Thank you for bringing these points to our attention.

**# Comment 5.** There are small typographic errors throughout the manuscript, that can be easily corrected. However, in the results section, the range of the population was 31-92 years. How can that be if the inclusion criteria was 60 or more years old? 6. MA is addressed in a not specific manner in this section. No analysis is presented concerning differences between gender, age group, pharmacologic class, diseases... this can influence your conclusions.

**# Author’s Response:** We appreciate the feedback provided. Regarding the typographical errors, we carefully reviewed the manuscript to correct these errors. Regarding the discrepancy in the age range, it appeared to be an oversight, and we apologize for any confusion it may have caused. We ensured to clarify this discrepancy in the revised version of the manuscript. Regarding the analysis of medication adherence (MA), we acknowledged the importance of considering various factors that could influence adherence, such as gender, age group, pharmacologic class, and diseases. We have certainly considered for incorporating your recommendations into our future projects to enhance the quality and comprehensiveness of our analyses. Your insights are valuable and will help us improve our research methodology and findings.

**# Comment 7.** Finally, you state that "The author(s) received no specific funding for this work", but at the end of the manuscript you write that there was a funding from the King Khalid University.

**Author’s Response:** Thank you for bringing this to our attention. We have corrected this inconsistency in the manuscript.

**# Comment 6.** PLOS authors have the option to publish the peer review history of their article (what does this mean?). If published, this will include your full peer review and any attached files.

**# Author’s Response:** Thank you for bringing this to our attention. Publishing the peer review history means making the reviews and any attached files provided by the reviewers available to the public alongside the published article. This allows readers to see the feedback and criticisms received during the peer review process, providing transparency and insight into the evaluation of the manuscript.
