# Supplementary material for: Medication adherence and quality of life among geriatric patients: Insights from a hospital-based cross-sectional study in India
Source: PLoS One. 2024 Nov 12;19(11):e0302546. doi: 10.1371/journal.pone.0302546 (PMC11556742; doi:10.1371/journal.pone.0302546)
Supplement: Attachment — Submitted filename: Reviewer Comments.docx [file pone.0302546.s002.docx]

**Reviewer Comments**

**General**

This manuscript needs a thorough review of the language to improve grammatical, syntax and spelling errors. The thematic linkages must be improved also.

**Introduction**

- The section has a lot of language difficulties which must be improved
- Line 2-4. The sentence *“…Based on the data from the 2001 census, it was seen that the population of individuals classified as elderly to approximately 77 million.”* must be reconstructed to make it clearer.
- “Cardiovascular disease” must not start with a capital letter
- The spelling of ‘ageing’ vs aging should be consistent

**Methodology**

- The section has a lot of language difficulties which must be improved
- Under ‘Screening of patients”, kindly check the spellings of ‘in patient’ and ‘out patient’, and keep it consistent throughout the manuscript. These occur in other sections. Also check word repetitions e.g. ‘patients patients.

**Results**

The descriptions should be improved.

**Discussion**

The language and linkages must be improved.
